# Setting the Inlay

**Published:** 1906-03

**Authors:** J. S. Bridges

**Affiliations:** Chicago.


### SETTING THE INLAY.

By J. S. Bridges, D. D. S., Chicago.

After two years of further practice, I find such favorable results attained, after the performance of setting many inlays, that I make no material difference in my method of setting porcelain inlays, from that set forth in this journal of July, 1904. I will therefore simply give that same article with some small additions and corrections.

Assuming our finished inlay has been tried in the cavity, and the test is to our liking, we are ready to seat the restoration into the waiting cavity.

*Preparation.*—Lay out the mixing slab, chilling it if the weather is excessively warm. Place upon one corner a small heap of powder and opposite a bit of liquid, dropped from the bottle. A medium sized German silver spatula should be laid near by. Invert the inlay upon a bed of yellow beeswax, and with an orange wood stick put a drop of hydrofluoric acid on the upturned surface, previously observing that the margins are securely sealed to guard the glazed surface from injury. Allowing the acid to remain two or three minutes, wash off with water. With compressed air, alcohol and a fine pointed instrument, remove every particle of powdered porcelain from the inlay. By means of absorbent cotton rolls, linen napkins, spunk, alcohol and compressed air, the cavity is isolated and thoroughly dried. The rubber dam is not advised except in rare cases, for by far too much danger is incurred from its tendency to buckle at the margins or become sticky from surplus cement, entangling the fingers or instruments; besides few cervical cavities will permit the use of a clamp or ligatures without extreme discomfort to the patient and considerable physical exertion to the operator.

Birds-eye linen cut in squares of about six inches make convenient sized napkins. Turn down one corner about an inch then fold to the center from the right and left and a splendid and comfortable dam for the superior teeth is formed. Cotton rolls give most excellent service about the inferior teeth and for banking against the salivary ducts when the flow is over copious. Spunk is an admirable assistant for drying the cavity and may be used to advantage if left in the cavity to absorb any secretions during the mixing of the cement.

*Selecting Cement Colors.*—A large variety of shades is a snare. Learn to work with as few as seems practicable. I use only three, pearl grey, yellow and white. Any one of these judiciously selected, or the blending of one with the other will allow sufficient latitude of color and make the choosing of the background less confusing. The tendency should be light rather than dark, but care must be taken that your cement is not so light that it shows through when the enamel wall is thin.

*Mixing the Cement.*—Bring the powder gradually, a small portion at a time, into the liquid, stirring one way until a thin cream is formed that just absorbs all of the liquid. Take sufficient of this on the point of the spatula to spread over the floor of the cavity and the base of the inlay. This done, continue adding powder to the mixture until the cream is so heavy you dare not add more for fear of losing the necessary adhesiveness. Place enough of this in the cavity to fill it to a generous overflow.

*Seating the Inlay.*—Quickly grasp the inlay with a pair of pliers and gently coax the filling over, but not completely into, its proper seat. Now, with another pair of pliers (the first are most likely gummed with cement) pick out a bit of spunk and press the inlay thoroughly home. The thin spread of cement by this time having nicely soaked into the tubuli of the cementum and the crevices of the porcelain, will act as a flux to the heavier cement sandwiched between and should allow the inlay to find its proper seat with but little strain. This arrangement of the cement will overcome many disasters from an insufficiently dense body, the lack of which permits the fluids to soon undermine and cause an otherwise perfect restoration to fail.

A simple mix may make the seating somewhat easier but will leave little powder behind as the over abundance of liquid carries most of it away when the inlay is forced into place.

Frequently in seating anterior fillings there is not enough space to readily manipulate the spunk. When such is the case a cuttle fish strip will be found of excellent service. Slip the tape between the inlay and the approximal tooth and with the thumb and forefinger hold the end tight against some adjacent tooth upon the side the inlay has been inserted. With the other hand draw the tape taut and gently force the filling into place. To overcome the expansion of the cement during the process of setting, the inlay should be kept under pressure for at least five or ten minutes before allowing the moisture to reach it. When this is out of the question permit a large amount of cement to remain about the margins, possibly covering the same with cement varnish, or melted *parafin* which may be cleared off at the next appointment.

It should seldom be necessary to polish or grind any surface but the occlusal. A smooth strip at the cervical margin is frequently beneficial, and when used should be followed by a bit of whiting placed under the strip, which will nicely bring back a safe surface to the products our best manufacturers have placed on the market.

